# Imported malaria from land bordering countries in China: A challenge in preventing the reestablishment of malaria transmission

**DOI:** 10.1016/j.tmaid.2023.102575

**Published:** 2023

**Authors:** Jian-hai Yin, Li Zhang, Bo-yu Yi, Shui-sen Zhou, Zhi-gui Xia

**Affiliations:** National Institute of Parasitic Diseases, Chinese Center for Disease Control and Prevention (Chinese Center for Tropical Diseases Research), NHC Key Laboratory of Parasite and Vector Biology, WHO Collaborating Center for Tropical Diseases, National Center for International Research on Tropical Diseases, Shanghai, 200025, China

**Keywords:** Imported malaria, Bordering country, *Plasmodium vivax*, Recurrence, Reestablishment, China

## Abstract

**Objective:**

No indigenous malaria cases have been reported since 2017 in China, but a large number of imported cases are still reported every year, including those from the land bordering countries. To characterize their epidemiological profiles will provide evidence for the development of appropriate strategies to effectively address the challenges of border malaria in the post-elimination phase.

**Methods:**

Individual-level data of imported malaria cases from the land bordering countries were collected from 2017 to 2021 in China via the web-based surveillance systems, and analyzed by SPSS, ArcGIS and WPS software, to explore their epidemiological profiles.

**Results:**

A total of 1170 malaria cases imported into China from six of the fourteen land bordering countries were reported between 2017 and 2021 with a decline trend. Overall, cases were widely distributed in 31–97 counties from 11 to 21 provinces but mainly in Yunnan. Moreover, these imported cases were mainly infected with *P. vivax* (94.8%), and a total of 68 recurrent cases were reported in 6–14 counties from 4 to 8 provinces. In addition, nearly 57.1% of the total reported cases could seek healthcare within 2 days of getting sick, and 71.3% of the reported cases could be confirmed as malaria on the day they sought medical care.

**Conclusions:**

China still needs to attach great importance to the risk and challenge of the imported malaria from bordering countries particularly from Myanmar in preventing reestablishment of malaria transmission in the post-elimination phase. It is necessary not only to strengthen collaboration and cooperation with the bordering countries, but also coordinate multiple departments at home to improve malaria surveillance and response system and prevent the reestablishment of malaria transmission in China.

## 1 Introduction

Malaria is still one of the major public health threats worldwide that cause a large number of cases and deaths, there were an estimated 247 million malaria cases in 84 malaria endemic countries and 619000 malaria deaths in 2021, and most of them occurred in the World Health Organization (WHO) African Region [1]. Fortunately, increasing countries were certified as malaria-free or approaching to malaria elimination. In China, malaria burden has been greatly reduced from 30 million cases per year in the 1940s to zero indigenous case in 2017 with continuous efforts in the fight against malaria [2], and the WHO officially declared China malaria-free on June 30, 2021 [3].

However, China still faces many challenges in the prevention of reestablishment of malaria transmission. First, high burden of imported malaria cases persists for many years. For example, a total of 10085 imported malaria cases were reported in China from 2017 to 2021 [[4], [5], [6], [7], [8]], which were mainly from Africa (86.7%) and Asia (12.4%). Those cases from Africa were mainly infected with *Plasmodium falciparum* (74.0%), and those from Asia were mainly infected with *P. vivax* (93.1%) [9]. Second, the challenge of border malaria cannot be neglected, even during the Coronavirus disease 2019 (Covid-19) pandemic with the travel restrictions. Globally, it is more difficult to control and eliminate malaria transmission in border areas, due to the specific environmental (including physical, social and geopolitical), anthropological, administrative and geographic characteristics [10]. China shares the longest land border in the world with 14 countries [11], including Afghanistan, Bhutan, India, Kazakhstan, Kyrgyzstan, Laos, Mongolia, Myanmar, Nepal, North Korea, Pakistan, Russia, Tajikistan, and Vietnam, and 9 countries are still malaria-endemic except for Kazakhstan, Kyrgyzstan, Mongolia, Russia, and Tajikistan [1]. Third, the risk of malaria resurgence caused by the imported cases remains in China, especially due to the dominated species of *P. vivax* in most bordering countries [1,12,13] and the extensive distribution of malaria vector *Anopheles* mosquitoes in China, particularly the *An. sinensis* is suitable for transmitting *P. vivax*. Significantly, introduced vivax malaria cases have occurred in elimination phase in Dandong of Liaoning Province [14], Longhui of Hunan Province [5,15], and Tengchong of Yunnan Province [16] in China, and all the three events were related to the imported cases from South-East Asia. In addition, the malaria surveillance and response system with the 1-3-7 approach as the core will be challenged in the post-elimination phase, because the awareness of malaria prevention and control may be weakened, and some people mistakenly think that the reestablishment of malara transmission can be achieved relatively easily with less funding and human resources, etc. [9]. Meanwhile, the awareness of healthcare seeking of malaria patients, and the vigilance and ability of front-line health providers to diagnose and treat malaria, as well as the capacity of laboratory testing and review, need to be further improved [9]. Therefore, it is necessary to intensify the surveillance and management of population to and from malaria endemic areas, particularly from the bordering countries, and understand the epidemiological characteristics for developing appropriate prevention strategies and measures.

In the present study, the epidemiological characteristics of malaria cases reported in China from the land bordering countries in the five years (2017–2021) are analyzed, which is intended to provide evidence for China to effectively address the challenges of border malaria in the post-elimination phase, and contribute to the prevention of malaria reestablishment in China.

## 2 Methods

### 2.1 Data collection

Data on malaria cases reported in Chinese mainland (excluding Hong Kong, Macao, and Taiwan) from the bordering countries from January 1, 2017, to December 31, 2021, were extracted from the China Information System for Disease Control and Prevention and Parasitic Diseases Information Reporting Management System. Variables included case classification, demographic information (age, gender, occupation), origins of malaria cases (bordering countries), *Plasmodium* species, and reported locations at provincial and county level.

All the cases were diagnosed and confirmed by microscopy, rapid diagnostic tests, or polymerase chain reaction in the malaria diagnostic laboratory network, according to the Criteria on Diagnosis of Malaria (WS259-2015) in China [17].

### 2.2 Data analysis

The data was basically processed using WPS Office, and the geographical distribution at the county level was performed using ArcGIS 10.8 (Esri Inc., Redlands, CA, USA) [18]. Then the comparative analysis between variables among different groups was conducted with Chi-square tests or Fisher's Exact Test using IBM SPSS Statistics (version 26). The level of significance was set at *P* < 0.05.

## 3 Results

**Fig. 1:**
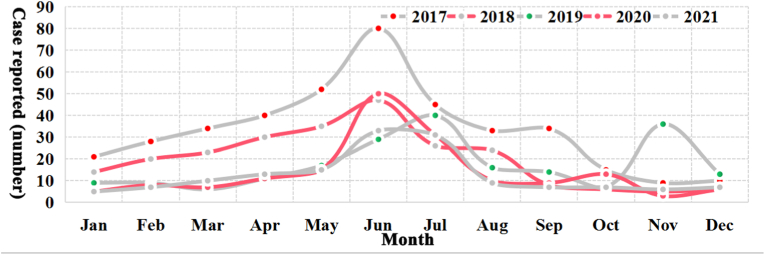
Temporal distribution of malaria cases from the bordering countries, 2017–2021.

From 2017 to 2021, a total of 1170 cases from six bordering countries (Myanmar, Pakistan, Laos, India, Vietnam, and Bhutan) were reported in China with a decreasing trend, and Myanmar (84.6%) was the most common source of imported cases, followed by Pakistan (11.1%), and there was a significant difference in the yearly composition of the source countries of imported cases (*P* < 0.001, Fisher's Exact Test) (Table 1). Moreover, cases have been reported every month in each year accompanied by a small peak in June usually (Fig. 1), the majority of reported cases each year were males (75.1%–81.5%) (χ^2^ = 3.531, *P* = 0.473), and the proportion of cases infected with *P. vivax* in each year has always exceeded 92%, and the remaining cases were infected with *P. falciparum* (0.6%–5.0%), *P. ovale* (0–1.9%), *P. malariae* (0–1.2%), and mixed species (0–1.6%). Meanwhile, there was a statistically significant difference in the age distribution of cases from year to year (χ^2^ = 26.215, *P* < 0.001), and most cases aged from 20 to 59 (82.7%, 968/1170). In addition, most cases were occurred in migrant workers (50.3%) and visitors who visited their relatives or friends (19.1%) (*P* < 0.001, Fisher's Exact Test) (Table 1).

**Table 1:** Demographic characteristics of malaria cases from the bordering countries reported in China, 2017–2021.

Demographic characteristics	Yearly number of cases (number, %)					Total
	2017	2018	2019	2020	2021	
Country						
Myanmar	315 (78.6)	205 (84.0)	173 (83.6)	160 (95.2)	137 (91.3)	990 (84.6)
Pakistan	66 (16.5)	26 (10.7)	21 (10.1)	6 (3.6)	11 (7.3)	130 (11.1)
Laos	13 (3.2)	5 (2.0)	4 (1.9)	1 (0.6)	1 (0.7)	24 (2.1)
India	5 (1.2)	7 (2.9)	5 (2.4)	1 (0.6)	0 (0)	18 (1.5)
Vietnam	1 (0.2)	1 (0.4)	4 (1.9)	0 (0)	1 (0.7)	7 (0.6)
Bhutan	1 (0.2)	0 (0)	0 (0)	0 (0)	0 (0)	1 (0.1)
*Plasmodium* species						
*Pf*	20 (5.0)	9 (3.7)	2 (1.0)	1 (0.6)	2 (1.3)	34 (2.9)
*Pv*	375 (93.5)	225 (92.2)	198 (95.7)	165 (98.2)	146 (97.3)	1109 (94.8)
*Po*	3 (0.7)	3 (1.2)	4 (1.9)	0 (0)	1 (0.7)	11 (0.9)
*Pm*	2 (0.5)	3 (1.2)	1 (0.5)	0 (0)	1 (0.7)	7 (0.6)
Mixed	1 (0.2)	4 (1.6)	2 (1.0)	2 (1.2)	0 (0)	9 (0.8)
Gender						
Male	301 (75.1)	192 (78.7)	163 (78.7)	137 (81.5)	119 (79.3)	912 (77.9)
Female	100 (24.9)	52 (21.3)	44 (21.3)	31 (18.5)	31 (20.7)	258 (22.1)
Age (years)						
<10	19 (4.7)	13 (5.3)	3 (1.4)	7 (4.2)	7 (4.7)	49 (4.2)
<20	36 (9.0)	19 (7.8)	11 (5.3)	16 (9.5)	14 (9.3)	96 (8.2)
<30	72 (18.0)	51 (20.9)	48 (23.2)	36 (21.4)	36 (24.0)	243 (20.8)
<40	98 (24.4)	61 (25.0)	53 (25.6)	47 (28.0)	21 (14.0)	280 (23.9)
<50	104 (25.9)	52 (21.3)	46 (22.2)	36 (21.4)	39 (26.0)	277 (23.7)
<60	55 (13.7)	32 (13.1)	35 (16.9)	20 (11.9)	26 (17.3)	168 (14.4)
≥60	17 (4.2)	16 (6.6)	11 (5.3)	6 (3.6)	7 (4.7)	57 (4.9)
Occupation						
Migrant workers	218 (54.4)	132 (54.1)	99 (47.8)	81 (48.2)	58 (38.7)	588 (50.3)
Visitors	98 (24.4)	48 (19.7)	38 (18.4)	29 (17.3)	10 (6.7)	223 (19.1)
Bussinessmen	19 (4.7)	16 (6.6)	15 (7.2)	5 (3.0)	3 (2.0)	58 (5.0)
Civil servants	4 (1.0)	3 (1.2)	6 (2.9)	1 (0.6)	4 (2.7)	18 (1.5)
Travelers	2 (0.5)	1 (0.4)	3 (1.4)	1 (0.6)	0 (0)	7 (0.6)
Others	60 (15.0)	44 (18.0)	46 (22.2)	51 (30.4)	75 (50.0)	276 (23.6)
Total	401	244	207	168	150	1170

### 3.1 Country of origin of malaria cases

From 2017 to 2021, the proportion of malaria cases from the bordering countries ranged from 7.7% (2019) to 18.8% (2021) of the total imported cases reported in China (Figure S1) [9]. However, vivax malaria cases from the bordering countries ranged from 57.3% (2018) to 80.2% (2021) of the total imported vivax malaria cases reported in China (Figure S1), and vivax malaria cases were also the most reported cases from each bordering country in every year (Table 2). Moreover, the majority of cases from these countries except for Bhutan were males (Table S1), and aged from 20 to 60 years old (Table S2).

**Table 2:** The profile of malaria cases from the bordering countries with different *Plasmodium* species, 2017–2021.

Country	Species	Yearly number of cases (number, %)					Total
		2017	2018	2019	2020	2021	
Myanmar	*Pf*	16 (5.1)	7 (3.4)	1 (0.6)	0 (0)	0 (0)	24 (2.4)
	*Pv*	297 (94.3)	190 (92.7)	171 (98.8)	159 (99.4)	136 (99.3)	953 (96.3)
	*Po*	1 (0.3)	1 (0.5)	1 (0.6)	0 (0)	0 (0)	3 (0.3)
	*Pm*	1 (0.3)	3 (1.5)	0 (0)	0 (0)	1 (0.7)	5 (0.5)
	Mixed	0 (0)	4 (2.0)	0 (0)	1 (0.6)	0 (0)	5 (0.5)
	Subtotal	315	205	173	160	137	990
Pakistan	*Pf*	3 (4.5)	1 (3.8)	0 (0)	1 (16.7)	2 (18.2)	7 (5.4)
	*Pv*	60 (90.9)	24 (92.3)	20 (95.2)	5 (83.3)	9 (81.8)	118 (90.8)
	*Po*	1 (1.5)	1 (3.8)	0 (0)	0 (0)	0 (0)	2 (1.5)
	*Pm*	1 (1.5)	0 (0)	0 (0)	0 (0)	0 (0)	1 (0.8)
	Mixed	1 (1.5)	0 (0)	1 (4.8)	0 (0)	0 (0)	2 (1.5)
	Subtotal	66	26	21	6	11	130
Laos	*Pf*	0 (0)	1 (20.0)	0 (0)	0 (0)	0 (0)	1 (4.2)
	*Pv*	12 (92.3)	4 (80.0)	2 (50.0)	1 (100.0)	0 (0)	19 (79.2)
	*Po*	1 (7.7)	0 (0)	1 (25.0)	0 (0)	1 (100.0)	3 (12.5)
	*Pm*	0 (0)	0 (0)	0 (0)	0 (0)	0 (0)	0 (0)
	Mixed	0 (0)	0 (0)	1 (25.0)	0 (0)	0 (0)	1 (4.2)
	Subtotal	13	5	4	1	1	24
India	*Pf*	1 (20.0)	0 (0)	1 (20.0)	0 (0)	0	2 (11.1)
	*Pv*	4 (80.0)	7 (100.0)	3 (60.0)	0 (0)	0	14 (77.8)
	*Po*	0 (0)	0 (0)	1 (20.0)	0 (0)	0	1 (5.6)
	*Pm*	0 (0)	0 (0)	0 (0)	0 (0)	0	0 (0)
	Mixed	0 (0)	0 (0)	0 (0)	1 (100.0)	0	1 (5.6)
	Subtotal	5	7	5	1	0	18
Vietnam	*Pf*	0 (0)	0 (0)	0 (0)	0	0 (0)	0 (0)
	*Pv*	1 (100.0)	0 (0)	2 (50.0)	0	1 (100.0)	4 (57.1)
	*Po*	0 (0)	1 (100.0)	1 (25.0)	0	0 (0)	2 (28.6)
	*Pm*	0 (0)	0 (0)	1 (25.0)	0	0 (0)	1 (14.3)
	Mixed	0 (0)	0 (0)	0 (0)	0	0 (0)	0 (0)
	Subtotal	1	1	4	0	1	7
Bhutan	*Pf*	0 (0)	0	0	0	0	0 (0)
	*Pv*	1 (100.0)	0	0	0	0	1 (100.0)
	*Po*	0 (0)	0	0	0	0	0 (0)
	*Pm*	0 (0)	0	0	0	0	0 (0)
	Mixed	0 (0)	0	0	0	0	0 (0)
	Subtotal	1	0	0	0	0	1
Total		401	244	207	168	150	1170

### 3.2 Spatial distribution of malaria cases in China

**Fig. 2:**
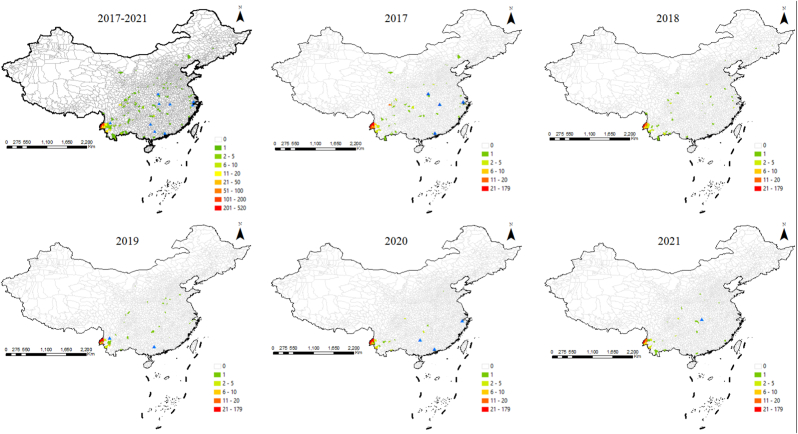
**The distribution of malaria cases from the bordering countries in China, 2017–2021.** Blue triangle means that cases were reported at the prefectural level. (For interpretation of the references to colour in this figure legend, the reader is referred to the Web version of this article.)

From 2017 to 2021, malaria cases from the bordering countries were reported in 194 counties from 25 provinces, including at least 31 (2020) to 97 (2017) counties from 11 (2020) to 21 (2018) provinces (Table S3), and most were reported in Yunnan from 73.3% (2017) to 88.7% (2020) (Table 3). In other words, the cases imported from Myanmar were reported in 22 provinces and mostly in Yunnan (90.9%, 900/990), and those from Pakistan were reported in 19 provinces (30 in Sichuan, 15 in Hubei, 12 in Henan, 11 in Zhejiang, 9 in Jiangsu and Shaanxi each, 8 in Hebei and Shandong each, 5 in Chongqing, 4 cases in Beijing, 3 in Jiangxi, Shanxi and Yunnan each, 2 in Guizhou and Hunan each, one in Anhui, Gansu, Jilin and Guangdong each), and cases from Laos were reported in 8 provinces (12 cases in Yunnan, 3 in Sichuan, 2 in Hubei, Hunan and Jiangsu each, and one in Gansu, Henan and Shandong each), and those from India were reported in 10 provinces (5 in Zhejiang, 4 in Guangdong, 2 in Jiangxi, one in Hebei, Chongqing, Jiangsu, Shandong, Shanghai, Shanxi and Guangxi each), and 7 cases from Vietnam were reported in 4 provinces (3 in Henan, 2 in Hunan, one in Jiangxi and Sichuan each), and one case from Bhutan was reported in Yunnan (Fig. 2).

**Table 3:** The distribution of malaria cases from the bordering countries at provincial level in China, 2017–2021.

Top level	Year (number, %)									
	2017		2018		2019		2020		2021	
**1**	Yunnan	294 (73.3)	Yunnan	180 (73.8)	Yunnan	163 (78.7)	Yunnan	149 (88.7)	Yunnan	127 (84.7)
**2**	Sichuan	35 (8.7)	Sichuan	12 (4.9)	Henan	9 (4.3)	Hunan	5 (3.0)	Sichuan	4 (2.7)
**3**	Zhejiang	12 (3.0)	Henan, Hubei	6 (2.5)	Zhejiang	5 (2.4)	Sichuan	5 (3.0)	Hunan	4 (2.7)
**4**	Others	60 (15.0)	Others	40 (16.4)	Others	30 (14.5)	Others	9 (5.4)	Others	15 (10.0)
**Total**		401		244		207		168		150

In terms of border malaria, there were a total of 13 bordering counties reported malaria cases from bordering countries, and all these counties are located in Yunnan, and most of the cases were from Myanmar (99.1%, 767/774) except for 6 cases from Laos and one from Bhutan in 2017 (Fig. 2). Moreover, ten bordering counties (Longyang and Tengchong in Baoshan, Lianghe, Longchuan, Mangshi, Ruili and Yingjiang in Dehong Dai and Jingpo Autonomous Prefecture, Jinping in Honghe Hani and Yi Autonomous Prefecture, Cangyuan and Gengma in Lincang) have reported cases for these five consecutive years (Fig. 2). In details, 250 cases were reported in 9 bordering counties in 2017, 155 cases in 12 bordering counties in 2018, 135 cases in 7 bordering counties in 2019, 126 cases in 8 bordering counties in 2020, and 108 cases in 8 bordering counties in 2021, respectively (Fig. 2).

**Fig. 3:**
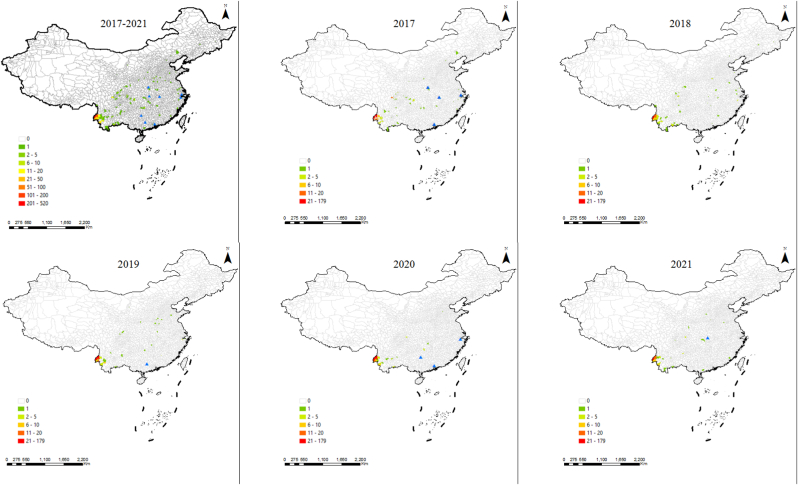
**The distribution of vivax malaria cases from the bordering countries in China, 2017–2021.** Blue triangle means that cases were reported at the prefectural level. (For interpretation of the references to colour in this figure legend, the reader is referred to the Web version of this article.)

In the case of vivax malaria, they were reported in 182 counties from 25 provinces, including 29 (2020) to 87 (2017) counties in 9 (2020) to 21 (2018) provinces (Fig. 3, Table S4), and Yunnan was also the province with the most reported cases in each year, with the proportion of 74.7% (280/375) in 2017, 75.1% (169/225) in 2018, 81.3% (161/198) in 2019, 90.3% (149/165) in 2020, and 86.3% (126/146) in 2021, respectively. Moreover, 97.4% (754/774) of cases reported in the 13 bordering counties were infected with *P. vivax*.

### 3.3 Healthcare seeking and case diagnosis

**Fig. 4:**
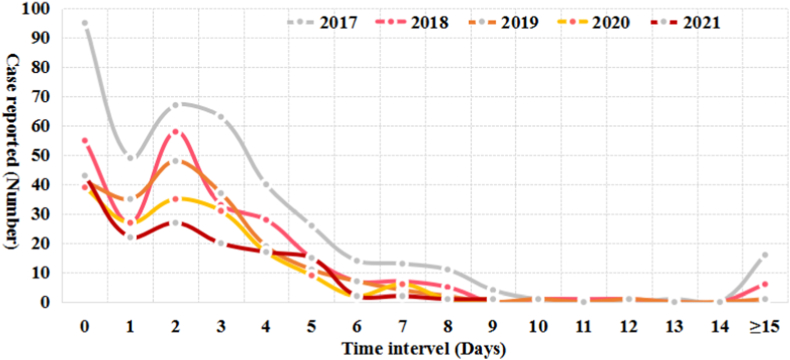
Time interval from onset to healthcare seeking among the malaria cases from the bordering countries reported in China, 2017–2021.

**Fig. 5:**
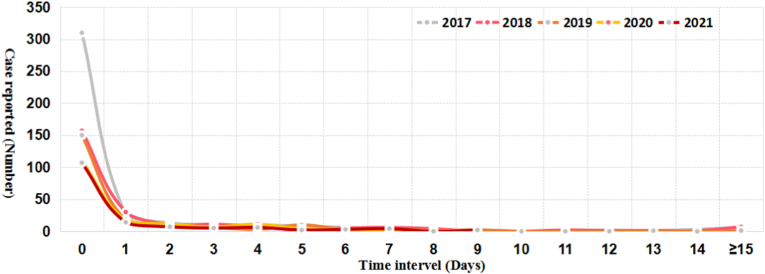
Time interval from healthcare seeking to diagnosis confirmation among the malaria cases from the bordering countries reported in China, 2017–2021.

The trend of the time interval from onset to healthcare seeking and from healthcare seeking to diagnosis confirmation was similar over a 5-year period respectively (Fig. 4, Fig. 5). Totally, only 23.3% of the reported cases could seek healthcare on the day (the interval is 0 day) they felt illness, and 13.7% of the total cases went to the clinics in the next day (the interval is 1 day), and 20.1% cases attended a doctor 2 days apart from the onset of illness (Fig. 4). In contrast, most (71.3%) of the reported cases could be confirmed as malaria on the day they sought medical care (Fig. 5).

### 3.4 Recurrent cases

A total of 68 recurrent cases were reported among the malaria cases originated from the bordering countries from 2017 to 2021, including 62 cases (91.2%) infected with *P. vivax* from Myanmar (41) and Pakistan (21), four cases (5.9%) of ovale malaria from Pakistan (2), Laos (1) and Vietnam (1), one case (1.5%) of falciparum malaria from Pakistan, and one case (1.5%) of mixed infections of *P. vivax* and *P. ovale* from Laos (Table S5). Among them, 88.2% cases were males (60/68), and 83.8% were aged from 20 to 50 years old (57/68) (Table S5), and 91.2% didn't receive radical treatment (62/68) except for 6 vivax malaria cases. Moreover, these cases were reported in 41 counties from 19 provinces, including 14 counties of 8 provinces in 2017, 12 counties of 8 provinces in 2018, 6 counties of 4 provinces in 2019, 7 counties of 3 provinces in 2020, and 12 counties of 7 provinces in 2021, respectively (Figure S2). In addition, the province with the highest number of recurrent cases reported was also Yunnan Province (44.1%, 30/68).

## 4 Discussion

In the present study, a total of 1170 malaria cases from six land bordering countries (Myanmar, Pakistan, Laos, India, Vietnam, and Bhutan) were reported from 2017 to 2021 with a decline trend in China. Cases were reported every month of each year, mainly infected with *P. vivax*, and widely distributed in 31 (2020) to 97 (2017) counties from 11 (2020) to 21 (2018) provinces, and most were reported in Yunnan (73.3%–88.7%), and all border counties where cases have been reported are also located in Yunnan Province. Moreover, the majority of cases were young and middle-aged male migrant labor workers, which was basically consistent with the situation of imported cases nationwide [[4], [5], [6], [7], [8]]. Additionally, a total of 68 recurrent cases (62 cases infected with *P. vivax*) were reported from 2017 to 2021 and distributed in 6 (2019) to 14 (2017) counties from 4 (2019) to 8 (2017, 2018) provinces.

China began to have no indigenous malaria cases reported in 2017 and was declared malaria-free by the WHO in 2021 [2,3], but a large number of imported cases were still reported every year [[4], [5], [6], [7], [8]]. In addition to a large number of cases from Africa, cases from malaria-endemic countries in Asia cannot be ignored. In the present study, it is found that about 11.6% (1170/10098) of cases were from countries bordering China [9], not only Myanmar, but also other neighboring countries such as Pakistan which still has a severe malaria situation in the near future [19]. Although the number of cases from neighboring countries has decreased due to the Covid-19 pandemic since 2020 (Table 1), its share of the number of reported cases in China has increased (Figure S1). Meanwhile, the proportion of cases from Myanmar has increased (Fig. 2), and their distribution within the country has shrunk, as well as the proportion in Yunnan has increased (Table 3). Second, malaria in Asian countries is dominated by *P. vivax* [1,12,13], and nearly 70.0% (1109/1671) of the vivax malaria cases reported in the country were from the neighboring countries in the past five years [9] (Table 1). Third, the China-Myanmar border remains a top priority [20,21], Myanmar is indeed the bordering countries with the largest source of imported malaria cases (Table 1), and Yunnan Province is the province with the most reported cases from these countries, of which 774 cases have been reported from 13 border counties in Yunnan, accounting for 84.8% (774/913) of reported cases in Yunnan, and most of the cases were from Myanmar (99.1%, 767/774) (Fig. 2, Table 3). Four, imported cases were also widely distributed in other parts of the country, especially in areas with *Anopheles sinensis* mosquitoes [22,23], which are widely distributed as suitable vectors for *P. vivax*, with a greater risk of reestablishment of transmission. In addition, it is worth noting that malaria including asymptomatic infections is still highly prevalent and there are always no natural barriers along the border [[24], [25], [26], [27]], and malaria infections caused by positive *Anopheles* mosquitoes that cross the border have been reported at a Chinese construction site on the China-Myanmar border [15], which are all important sources of infection [[28], [29], [30]]. If surveillance cannot detect such sources in time, they will be a greater risk of reestablishment of transmission in the post-elimination phase.

All countries that are malaria free should stop onward transmission from imported cases as a top priority, through sustaining the minimum activities necessary to maintain the sensitive malaria surveillance and response system against imported cases that might occur at any time and anywhere, and prevent reestablishment in areas with malariogenic potential [31]. The public awareness of malaria is one of the important prerequisites for the timely detection of malaria cases [32], but it is found that the timeliness of healthcare seeking is not high among the malaria cases from neighboring countries in the past five years, only 57.1% of the total reported cases could seek healthcare within 2 days of getting sick, and there were relatively high numbers of patients seeking healthcare after 15 days from onset of symptoms, which was an important risk that could lead to secondary transmission, as a large proportion of these cases infected with *P. vivax*. Fortunately, nearly 71.3% of cases were diagnosed as malaria at the first diagnosis, saving some time for the early detection of the source of infection. However, it is still recommended to strengthen health education for these populations and improve their malaria awareness, as well as strengthen clinicians’ malaria vigilance and diagnosis and treatment competency, to consolidate achievements in malaria elimination [33]. Especially for cases infected with *P. vivax* and/or *P. ovale*, it is necessary to strictly follow the requirements of guidelines for malaria diagnosis and treatment, and timely administrate radical treatment to avoid recurrence [[34], [35], [36]]. And 42 recurrent cases from the bordering countries between 2017 and 2021 were distributed in the historical malaria endemic provinces such as Yunnan, Sichuan, Zhejiang, Hubei, Henan and others (Figure S2), which is a potential risk of reestablishment.

## 5 Conclusions

China still needs to attach great importance to coordinate the collaboration and cooperation of multiple departments and fields at home and abroad, and effectively implement the strategies and measures of the Management Measures and Technical Guideline for prevention of reestablishment of malaria transmission in the postelimination phase [37,38]. Given that the imported malaria from neighboring countries will continue to pose an important challenge to maintain malaria free status in China, particularly in Yunnan Province, which borders 3 malaria-endemic countries: Myanmar, Laos and Viet Nam, the following points are worth considering. First of all, China not only needs to continue to focus on joint malaria prevention and control along the China-Myanmar border, but also strengthen cooperation and exchanges with other neighboring countries in the prevention and control of malaria and other infectious diseases, especially to strengthen malaria parasite screening for immigrants and border migrants [39], which will be a rebound following the lifting of travel restrictions due to the Covid-19 pandemic. Secondly, health education on malaria prevention and control should continue to be strengthened, especially for mobile populations traveling to and from malaria-endemic areas [32], in order to improve the timeliness of healthcare seeking. In addition, clinicians' vigilance and diagnosis and treatment capacity for malaria as well as the case investigation and disposal capacity must continue to be strengthened, so as to systematically improve malaria surveillance and response system after elimination and prevent the reestablishment of malaria transmission in China.

## Funding

This study was supported by the National Science and Technology Major Program of China, China (No. 2018ZX10101002–002-005), and the 10.13039/100000865Bill & Melinda Gates Foundation (No. INV-018913).

## Availability of data and materials

The datasets used and/or analyzed during the present study are available from the corresponding author upon reasonable request.

## Ethics approval and consent to participate

Not applicable.

## Consent for publication

Not applicable.

## CRediT authorship contribution statement

**Jian-hai Yin:** Conceptualization, Methodology, Formal analysis, Data curation, Writing – original draft, Writing – review & editing. **Li Zhang:** Data curation, Data collection and curation. **Bo-yu Yi:** Data curation, Data collection and curation. **Shui-sen Zhou:** Writing – review & editing. **Zhi-gui Xia:** Conceptualization, Writing – review & editing.

## Declaration of competing interest

The authors declare that they have no competing interests.
