# Supplementary material for: Imported malaria from land bordering countries in China: A challenge in preventing the reestablishment of malaria transmission
Source: Travel Med Infect Dis. 2023 May-Jun;53:102575. doi: 10.1016/j.tmaid.2023.102575 (PMC10250815; doi:10.1016/j.tmaid.2023.102575)
Supplement: Multimedia component 1 [file mmc1.docx]

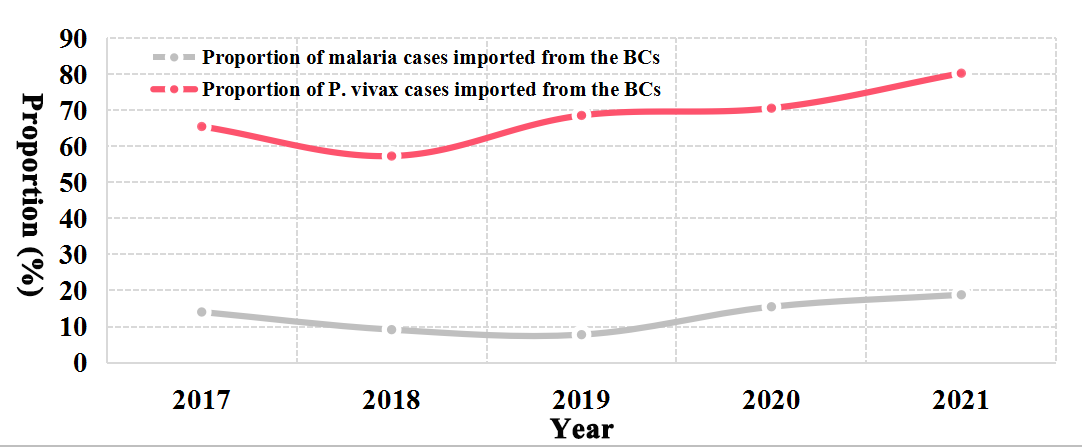


**Figure S1. The dynamics of imported cases from the bordering countries reported in China, 2017-2021**

**Figure S2. The distribution of recurrent malaria cases from the bordering countries reported in China, 2017-2021.** There were two cases originated from Myanmar were reported at the prefectural level: one vivax malaria case reported in Dongguan of Guangdong in 2020 and in Jingzhou of Hubei in 2021 each.
