# Supplementary material for: Imported malaria from land bordering countries in China: A challenge in preventing the reestablishment of malaria transmission
Source: Travel Med Infect Dis. 2023 May-Jun;53:102575. doi: 10.1016/j.tmaid.2023.102575 (PMC10250815; doi:10.1016/j.tmaid.2023.102575)
Supplement: Multimedia component 2 [file mmc2.docx]

**Table S1. The gender profile of malaria cases from the bordering countries, 2017-2021**

| **Country** | **Gender** | **Yearly number of cases (number, %)** | | | | | **Total** |
| --- | --- | --- | --- | --- | --- | --- | --- |
|  |  | **2017** | **2018** | **2019** | **2020** | **2021** |  |
| **Myanmar** | **Male** | 221 (70.2) | 155 (75.6) | 129 (74.6) | 130 (81.3) | 106 (77.4) | 741 (74.8) |
|  | **Female** | 94 (29.8) | 50 (24.4) | 44 (25.4) | 30 (18.8) | 31 (22.6) | 249 (25.2) |
| **Pakistan** | **Male** | 62 (93.9) | 24 (92.3) | 21 (100.0) | 6 (100.0) | 11 (100.0) | 124 (95.4) |
|  | **Female** | 4 (6.1) | 2 (7.7) | 0 (0) | 0 (0) | 0 (0) | 6 (4.6) |
| **Laos** | **Male** | 12 (92.3) | 5 (100.0) | 4 (100.0) | 1 (100.0) | 1 (100.0) | 23 (95.8) |
|  | **Female** | 1 (7.7) | 0 (0) | 0 (0) | 0 (0) | 0 (0) | 1 (4.2) |
| **India** | **Male** | 5 (100.0) | 7 (100.0) | 5 (100.0) | 0 (0) | 0 | 17 (94.4) |
|  | **Female** | 0 (0) | 0 (0) | 0 (0) | 1 (100.0) | 0 | 1 (5.6) |
| **Vietnam** | **Male** | 1 (100.0) | 1 (100.0) | 4 (100.0) | 0 | 1 (100.0) | 7 (100.0) |
|  | **Female** | 0 (0) | 0 (0) | 0 (0) | 0 | 0 (0) | 0 (0) |
| **Bhutan** | **Male** | 0 (0) | 0 | 0 | 0 | 0 | 0 (0) |
|  | **Female** | 1 (100.0) | 0 | 0 | 0 | 0 | 1 (100.0) |
| **Total** | | 401 | 244 | 207 | 168 | 150 | 1170 |

**Table S2. The age profile of malaria cases from the bordering countries, 2017-2021**

| **Country** | **Age (years)** | **Yearly number of cases (number, %)** | | | | | **Total** |
| --- | --- | --- | --- | --- | --- | --- | --- |
|  |  | **2017** | **2018** | **2019** | **2020** | **2021** |  |
| **Myanmar** | **<10** | 19 (6.0) | 13 (6.3) | 3 (1.7) | 7 (4.4) | 7 (5.1) | 49 (4.9) |
|  | **<20** | 35 (11.1) | 18 (8.8) | 9 (5.2) | 16 (10.0) | 14 (10.2) | 92 (9.3) |
|  | **<30** | 59 (18.7) | 40 (19.5) | 41 (23.7) | 34 (21.3) | 31 (22.6) | 205 (20.7) |
|  | **<40** | 71 (22.5) | 50 (24.4) | 41 (23.7) | 42 (26.3) | 17 (12.4) | 221 (22.3) |
|  | **<50** | 70 (22.2) | 44 (21.5) | 39 (22.5) | 36 (22.5) | 35 (25.5) | 224 (22.6) |
|  | **<60** | 46 (14.6) | 24 (11.7) | 29 (16.8) | 19 (11.9) | 26 (19.0) | 144 (14.5) |
|  | **≥60** | 15 (4.8) | 16 (7.8) | 11 (6.4) | 6 (3.8) | 7 (5.1) | 55 (5.6) |
| **Pakistan** | **<10** | 0 (0) | 0 (0) | 0 (0) | 0 (0) | 0 (0) | 0 (0) |
|  | **<20** | 0 (0) | 1 (3.8) | 1 (4.8) | 0 (0) | 0 (0) | 2 (1.5) |
|  | **<30** | 11 (16.7) | 7 (26.9) | 5 (23.8) | 2 (33.3) | 5 (45.5) | 30 (23.1) |
|  | **<40** | 20 (30.3) | 6 (23.1) | 8 (38.1) | 3 (50.0) | 3 (27.3) | 40 (30.8) |
|  | **<50** | 25 (37.9) | 7 (26.9) | 3 (14.3) | 0 (0) | 3 (27.3) | 38 (29.2) |
|  | **<60** | 9 (13.6) | 5 (19.2) | 4 (19.0) | 1 (16.7) | 0 (0) | 19 (14.6) |
|  | **≥60** | 1 (1.5) | 0 (0) | 0 (0) | 0 (0) | 0 (0) | 1 (0.8) |
| **Laos** | **<10** | 0 (0) | 0 (0) | 0 (0) | 0 (0) | 0 (0) | 0 (0) |
|  | **<20** | 1 (7.7) | 0 (0) | 0 (0) | 0 (0) | 0 (0) | 1 (4.2) |
|  | **<30** | 0 (0) | 1 (20.0) | 0 (0) | 0 (0) | 0 (0) | 1 (4.2) |
|  | **<40** | 4 (30.8) | 1 (20.0) | 1 (25.0) | 1 (100.0) | 0 (0) | 7 (29.2) |
|  | **<50** | 7 (53.8) | 0 (0) | 2 (50.0) | 0 (0) | 1 (100.0) | 10 (41.7) |
|  | **<60** | 0 (0) | 3 (60.0) | 1 (25.0) | 0 (0) | 0 (0) | 4 (16.7) |
|  | **≥60** | 1 (7.7) | 0 (0) | 0 (0) | 0 (0) | 0 (0) | 1 (4.2) |
| **India** | **<10** | 0 (0) | 0 (0) | 0 (0) | 0 (0) | 0 | 0 (0) |
|  | **<20** | 0 (0) | 0 (0) | 1 (20.0) | 0 (0) | 0 | 1 (5.6) |
|  | **<30** | 2 (40.0) | 2 (28.6) | 1 (20.0) | 0 (0) | 0 | 5 (27.8) |
|  | **<40** | 3 (60.0) | 4 (57.1) | 3 (60.0) | 1 (100.0) | 0 | 11 (61.1) |
|  | **<50** | 0 (0) | 1 (14.3) | 0 (0) | 0 (0) | 0 | 1 (5.6) |
|  | **<60** | 0 (0) | 0 (0) | 0 (0) | 0 (0) | 0 | 0 (0) |
|  | **≥60** | 0 (0) | 0 (0) | 0 (0) | 0 (0) | 0 | 0 (0) |
| **Vietnam** | **<10** | 0 (0) | 0 (0) | 0 (0) | 0 | 0 (0) | 0 (0) |
|  | **<20** | 0 (0) | 0 (0) | 0 (0) | 0 | 0 (0) | 0 (0) |
|  | **<30** | 0 (0) | 1 (100.0) | 1 (25.0) | 0 | 0 (0) | 2 (28.6) |
|  | **<40** | 0 (0) | 0 (0) | 0 (0) | 0 | 1 (100.0) | 1 (14.3) |
|  | **<50** | 1 (100.0) | 0 (0) | 2 (50.0) | 0 | 0 (0) | 3 (42.9) |
|  | **<60** | 0 (0) | 0 (0) | 1 (25.0) | 0 | 0 (0) | 1 (14.3) |
|  | **≥60** | 0 (0) | 0 (0) | 0 (0) | 0 | 0 (0) | 0 (0) |
| **Bhutan** | **<10** | 0 (0) | 0 | 0 | 0 | 0 | 0 (0) |
|  | **<20** | 0 (0) | 0 | 0 | 0 | 0 | 0 (0) |
|  | **<30** | 0 (0) | 0 | 0 | 0 | 0 | 0 (0) |
|  | **<40** | 0 (0) | 0 | 0 | 0 | 0 | 0 (0) |
|  | **<50** | 1 (100.0) | 0 | 0 | 0 | 0 | 1 (100.0) |
|  | **<60** | 0 (0) | 0 | 0 | 0 | 0 | 0 (0) |
|  | **≥60** | 0 (0) | 0 | 0 | 0 | 0 | 0 (0) |
| **Total** | | 401 | 244 | 207 | 168 | 150 | 1170 |

**Table S3. The distribution of malaria cases from the bordering countries in China, 2017-2021**

| **Administrative level** | **Yearly number** | | | | |
| --- | --- | --- | --- | --- | --- |
|  | **2017** | **2018** | **2019** | **2020** | **2021** |
| **Province** | 20 | 21 | 19 | 11 | 13 |
| **County** | 97* | 73 | 52* | 31* | 35* |

*There were 4, 2, 3 and 1 prefectural CDC reported 5, 2, 3 and 1 case in 2017, 2019, 2020 and 2021, respectively.

**Table S4. The distribution of *P. vivax* malaria cases from the bordering countries in China, 2017-2021**

| **Administrative level** | **Yearly number** | | | | |
| --- | --- | --- | --- | --- | --- |
|  | **2017** | **2018** | **2019** | **2020** | **2021** |
| **Province** | 19 | 21 | 17 | 9 | 13 |
| **County** | 87* | 67 | 46* | 29* | 32* |

*There were 4, 2, 2 and 1 prefectural CDC reported 5, 2, 2 and 1 case in 2017, 2019, 2020 and 2021, respectively.

**Table S5.** **The profile of recurrent cases derived from the bordering countries, 2017-2021**

| **Country** | **Groups** | **Yearly number of cases** | | | | | **Total** |
| --- | --- | --- | --- | --- | --- | --- | --- |
|  |  | **2017** | **2018** | **2019** | **2020** | **2021** |  |
| **Myanmar** | ***Pv*** | 5 | 10 | 3 | 9 | 14 | 41 |
|  | **Male** | 5 | 8 | 1 | 7 | 13 | 34 |
|  | **Female** | 0 | 2 | 2 | 2 | 1 | 7 |
|  | **<10** | 0 | 0 | 0 | 0 | 1 | 1 |
|  | **<30** | 2 | 4 | 2 | 1 | 3 | 12 |
|  | **<40** | 1 | 5 | 0 | 6 | 1 | 13 |
|  | **<50** | 1 | 0 | 0 | 1 | 7 | 9 |
|  | **<60** | 1 | 1 | 0 | 1 | 2 | 5 |
|  | **≥60** | 0 | 0 | 1 | 0 | 0 | 1 |
| **Pakistan** | ***Pf*** | 1 | 0 | 0 | 0 | 0 | 1 |
|  | ***Pv*** | 13 | 3 | 3 | 0 | 2 | 21 |
|  | ***Po*** | 1 | 1 | 0 | 0 | 0 | 2 |
|  | **Male** | 14 | 4 | 3 | 0 | 2 | 23 |
|  | **Female** | 1 | 0 | 0 | 0 | 0 | 1 |
|  | **<20** | 0 | 1 | 0 | 0 | 0 | 1 |
|  | **<30** | 3 | 1 | 0 | 0 | 1 | 5 |
|  | **<40** | 3 | 1 | 3 | 0 | 1 | 8 |
|  | **<50** | 6 | 1 | 0 | 0 | 0 | 7 |
| **Laos** | ***Po*** | 0 | 0 | 0 | 0 | 1 | 1 |
|  | **Mixed** | 0 | 0 | 1 | 0 | 0 | 1 |
|  | **Male** | 0 | 0 | 1 | 0 | 1 | 2 |
|  | **Female** | 0 | 0 | 0 | 0 | 0 | 0 |
|  | **<50** | 0 | 0 | 1 | 0 | 1 | 2 |
| **Vietnam** | ***Po*** | 0 | 1 | 0 | 0 | 0 | 1 |
|  | **Male** | 0 | 1 | 0 | 0 | 0 | 1 |
|  | **Female** | 0 | 0 | 0 | 0 | 0 | 0 |
|  | **<30** | 0 | 1 | 0 | 0 | 0 | 1 |
| **Total** | | 20 | 15 | 7 | 9 | 17 | 68 |
